# Effect of Food on the Steady-State Pharmacokinetics of Tenofovir and Emtricitabine plus Efavirenz in Ugandan Adults

**DOI:** 10.1155/2012/105980

**Published:** 2012-02-20

**Authors:** Mohammed Lamorde, Pauline Byakika-Kibwika, William S. Tamale, Francis Kiweewa, Mairin Ryan, Alieu Amara, John Tjia, David Back, Saye Khoo, Marta Boffito, Cissy Kityo, Concepta Merry

**Affiliations:** ^1^Infectious Diseases Institute, Makerere University, College of Health Sciences, P.O. Box 22418, Kampala, Uganda; ^2^Department of Pharmacology and Therapeutics, Trinity Centre for Health Sciences, St James's Hospital, Dublin 8, Ireland; ^3^Infectious Diseases Network for Treatment and Research in Africa, Plot 537, Tufnell Drive, Mulago Hill, P.O. Box 70345, Kampala, Uganda; ^4^Joint Clinical Research Centre, P.O. Box 10005, Kampala, Uganda; ^5^Department of Molecular and Clinical Pharmacology, University of Liverpool, Liverpool L69 3GF, UK; ^6^St Stephen's Centre, Chelsea and Westminster Hospital, 369 Fulham Road, London SW10 9NH, UK; ^7^Department of Genitourinary Medicine and Infectious Diseases, St James's Hospitals, Dublin 8, Ireland

## Abstract

We investigated the effect of food on the steady-state pharmacokinetics of a proprietary fixed-dose combination (FDC) tablet containing tenofovir disoproxil fumarate (TDF)/emtricitabine/efavirenz. Fifteen Ugandan HIV-1 patients at steady-state dosing with TDF/emtricitabine/efavirenz were admitted for 24-hour intensive pharmacokinetic sampling after dosing in the fasting state. Blood sampling was repeated seven days later with TDF/emtricitabine/efavirenz administered with food (19 g fat). Drug concentrations in plasma were determined by liquid chromatography and tandem mass spectrometry. Geometric mean ratios (GMRs) and confidence intervals (CIs) of parameters were calculated (reference, fasting). For efavirenz, GMRs (90% CIs) for *C*_max_, AUC_0−24_, and *C*_24_ were 1.47 (1.24–1.75), 1.13 (1.03–1.23), and 1.01 (0.91–1.11), respectively. Corresponding GMRs were 1.04 (0.84–1.27), 1.19 (1.10–1.29), and 0.99 (0.82–1.19) for tenofovir, 0.83 (0.76–0.92), 0.87 (0.78–0.97), and 0.91 (0.73–1.14) for emtricitabine. Stable patients may take the FDC without meal restrictions. The FDC should be taken without food by patients experiencing central nervous system toxicities.

## 1. Introduction

Food intake around the time of drug dosing may alter the bioavailability of orally administered drugs, and food effects can vary from drug to drug [1]. For fixed-dose combinations (FDCs), food intake may have an unbalanced impact on the component drugs and consensus must be achieved on the meal condition which results in optimal drug exposure for the overall regimen. Tenofovir disoproxil fumarate (TDF) and emtricitabine plus efavirenz is recommended for first-line treatment of HIV-1-infected adults [2–4]. These drugs were released as a single FDC tablet in 2006 following demonstration of bioequivalence between the FDC and the individual dosage forms [5].

Prior to the release of the FDC formulation, manufacturers of individual formulations had issued divergent guidance on food intake during drug dosing. In a single-dose study, a high-fat meal increased exposure (area under the curve, AUC) of TDF by 40% leading to a recommendation to dose TDF along with a meal [6]. Similarly, a high-fat meal increased AUC and maximal concentrations (*C*_max⁡_) of efavirenz by 28% and 79%, respectively. In this instance, the manufacturer recommended that efavirenz be administered without food because elevated efavirenz concentrations may lead to an increased frequency of adverse events [7]. Emtricitabine exposures were unaffected by food intake; therefore, the manufacturer recommended that it could be taken without regard to food [8].

Currently, the manufacturers of the FDC recommend that the tablet be administered without food. However, the new tablet formulation has not been formally evaluated in the presence of food [9]. Ugandan antiretroviral guidelines prefer drugs that are administered without food restrictions [4]. In Uganda, certain patients may prefer to dose their drugs with food because they believe that all antiretroviral drugs must be taken with food to prevent side effects [10]. Although this belief is unfounded, regimens which must be administered without food may be less acceptable to this group of patients.

In order to determine if the FDC can be administered with food, the current study compared the steady-state pharmacokinetics of tenofovir, emtricitabine, and efavirenz during administration of a proprietary FDC containing TDF and emtricitabine plus efavirenz in the fasting state or with a moderate-fat meal in HIV-1-infected Ugandan adults.

Western meals used in food effect studies can greatly differ from meals consumed in African settings, and pharmacokinetic data are scarce in African patients receiving local meals. Therefore, the present study was conducted using a local Ugandan meal.

## 2. Methods

### 2.1. Ethics and Regulatory Approval

Ethics committee approval was granted by the Joint Clinical Research Centre (JCRC) Institutional Review Board (Study code: JAFS). The trial was registered with the UNCST (HS553) and on http://www.pactr.org/ (PACTR2009120001702102).

### 2.2. Study Design

HIV-1-infected patients receiving TDF and emtricitabine plus efavirenz (Atripla, Bristol Myers Squibb & Gilead Sciences LLC) one tablet daily for greater than one month were recruited into this open-label, two-phase, single-sequence, cross-over pharmacokinetic study.

### 2.3. Patients

The study was conducted at JCRC Mengo, Kampala. Patients were enrolled if they provided written informed consent, were between 18 and 65 years of age, and had no recent use of medications (including traditional medicines) known to interfere with cytochrome P450 (CYP) metabolism. Patients were excluded if they were anaemic (serum haemoglobin < 10 g/dL), had significant derangement in renal function (serum creatinine > 3.4 mg/dL) or hepatic function (serum alanine transaminase > 5 times the upper limit of normal), or if they had severe intercurrent illnesses, vomiting, or diarrhoea or were unable to adhere to the prescribed meal sequence of the study. Women were excluded if they were pregnant.

### 2.4. Pharmacokinetic Sampling

Enrolled patients attended two 24-hour intensive pharmacokinetic sampling visits scheduled one week apart (day 1 and day 8). Patients taking their antiretroviral drugs at night were switched to morning dosing of a minimum of 3 days before the first sampling visit. On the morning of Day 1 (fasting), one FDC tablet of TDF and emtricitabine plus efavirenz (300 mg/200 mg/600 mg, resp.) was administered to patients after an overnight fast. Blood samples were collected before dosing and at 0.5, 1, 2, 3, 4, 6, 8, 12, and 24 hours after dosing. Breakfast was provided 3-4 hours post-dosing (see below for details). At each time point, 4 mL of venous blood was collected into ethylenediaminetetraacetic-acid-containing tubes, and samples were transferred to the JCRC laboratory within 1 hour of collection. Blood samples were centrifuged at 1500 g for 10 minutes to obtain plasma. Plasma obtained during centrifugation was transferred into 2 mL cryovials and stored at −70°C pending sample shipment. After collection of the blood sample at the 24-hour time-point, patients were allowed home and scheduled for a repeat pharmacokinetic evaluation one week later.

On Day 8 patients were readmitted after an overnight fast and given a standardized moderate fat Ugandan meal. The meal contained 650 Kcal and was composed of approximately 19 g of fat. The meal contents included matooke (local bananas, cooked vegetables, oil and meat, and tea with milk). Meals were started and completed within the 30 minutes prior to the scheduled time of TDF and emtricitabine plus efavirenz dosing. Blood sampling and plasma processing were conducted as in Day 1.

### 2.5. Determination of Tenofovir, Emtricitabine, and Efavirenz

The laboratory phase was conducted at the Department of Molecular and Clinical Pharmacology, University of Liverpool. Plasma samples were pretreated at 58°C for 40 minutes to inactivate HIV-1 and other pathogens. Tenofovir and emtricitabine were isolated from plasma by protein precipitation and solid-phase extraction, and concentrations were determined using a triple quadrupole liquid chromatography with tandem mass spectrometry (LC-MS/MS). Analytes were resolved on a phenomenex Synergi Polar C_18_ reverse phase 4 *μ* (150 × 2 mm) column. The lower limit of quantification (LLOQ) for tenofovir was 5.4 ng/mL, accuracy ranged from 86.0 to 95.1%, and imprecision was below 16.5%. The LLOQ for emtricitabine was 6.8 ng/mL, accuracy ranged from 95.5 to 101.0%, and imprecision was below 11.3%.

Efavirenz was isolated from plasma by protein precipitation, and the concentration was determined using LC-MS/MS. Hexobarbital was used as the internal standard. The analytic column was an Atlantis *C*_18_ reverse phase 3 *μ* (50 × 2.1 mm) LC column. The LLOQ for efavirenz was 8.5 ng/mL. Assay accuracy ranged from 93.2 to 101.8%, and imprecision was below 7.5%.

The laboratory participates in an external quality control program for antiretroviral drugs/(http://www.kkgt.nl/)

### 2.6. Safety Assessments

Reported adverse events were recorded at pharmacokinetic sampling visits.

### 2.7. Data Analysis

Patient demographic parameters are presented as summary statistics (medians, interquartile ranges). Pharmacokinetic parameters including maximal concentrations (*C*_max⁡_), time to *C*_max⁡_ (*T*_max⁡_), and concentrations 24 hours after dosing (*C*_24_) were obtained from the data. The AUC over 24 hours (AUC_0−24_) and half-life (*t*_1/2_) were calculated by noncompartmental methods (WinNonlin Version5.2, Pharsight, MountainView, CA, USA). Values for *T*_max⁡_ are presented as medians and interquartile ranges.

Geometric means (GMs) and ratios (GMRs) were calculated for AUC_0−24_, *C*_max⁡_, and *C*_24_. The fasting state (Day 1) was used as the reference based on the manufacturer's recommendation to dose the FDC without food. Absence of a food effect was assumed if the 90% CI for GMR between fed and fasting treatments was not contained in the equivalence limits of 80–125% for either AUC_0−24_ or *C*_max⁡_. Tenofovir and emtricitabine *t*_1/2_ was presented as harmonic means and 90% CI. The 24-hour dosing interval is inadequate to characterise efavirenz *t*_1/2_, and these results are not presented. Efavirenz plasma concentrations 12 hours after dosing (*C*_12_) were evaluated using the suggested therapeutic range of 1,000–4,000 ng/mL based on a study in which efavirenz samples were collected between 8 and 20 hours after dosing [11].

## 3. Results

### 3.1. Patients

Demographic characteristics of patients at screening are shown in Table 1. Of the 15 patients enrolled, 11 were male. At enrolment, patients were on treatment with the TDF and emtricitabine plus efavirenz FDC for a median (interquartile range) of 413 (210–600) days. Fourteen patients were receiving cotrimoxazole prophylaxis while one patient was on dapsone. All 15 patients completed the study.

### 3.2. Pharmacokinetics

**Figure 1:**
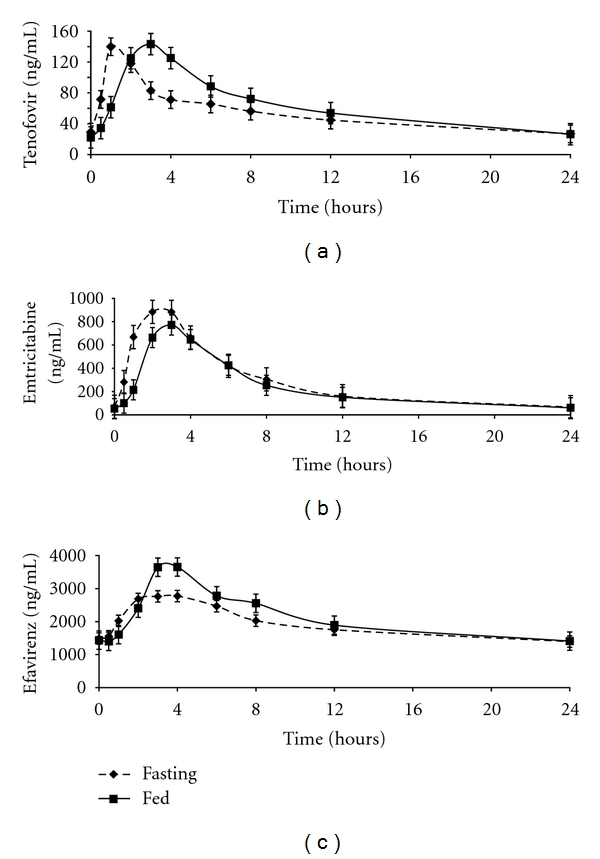
24-hour plasma-concentration-time profiles of (a) tenofovir, (b) emtricitabine, and (c) efavirenz when administered in the fed and fasting states. Error bars, standard error.

**Figure 2:**
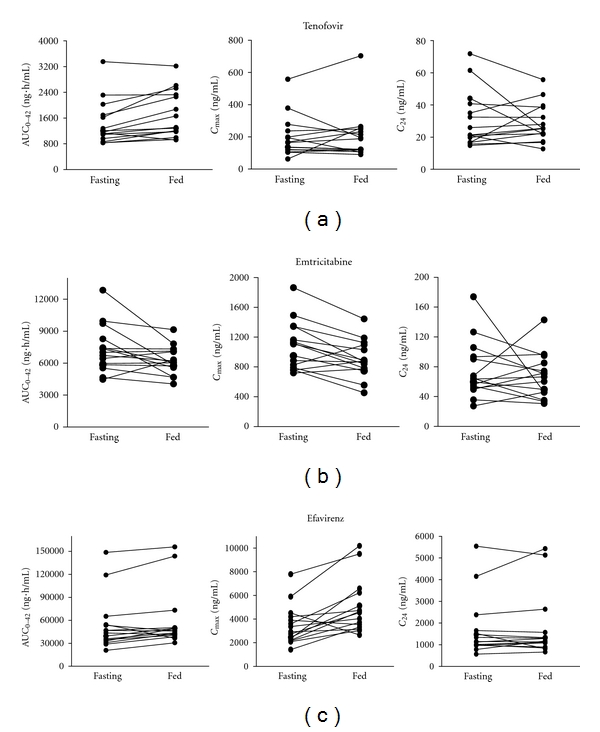
Individual pharmacokinetic parameters for (a) tenofovir and (b) emtricitabine when administered in the fed and fasting states. (c) Individual pharmacokinetic parameters for efavirenz when administered in the fed and fasting states.

Figure 1 shows 24-hour plasma-concentration-time profiles for tenofovir, emtricitabine, and efavirenz under fed and fasting dosing conditions. Plasma pharmacokinetic parameters and comparisons for these three drugs are presented in Table 2.  Figure 2 displays individual AUC_0−24_, *C*_max⁡_, and *C*_24_ of the study drugs when administered under fasting and fed conditions.

Median (interquartile range) tenofovir *T*_max⁡_ was 2.0 (1.5–3.0) hours in the fasting state and 3.0 (2.0-3.0) hours with food. Tenofovir AUC_0−24_ was significantly increased by 19% in the presence of food while other parameters were not significantly altered by food intake. In the fasting and fed states, CVs for AUC_0−24_ was 48% and 43%; *C*_max⁡_ was 65% and 74%; *C*_24_ was 57% and 41%, respectively. Other parameters were not significantly altered by the presence of food. On Day 1 and Day 8, *t*_1/2_ was 10.3 (9.8–12.1) and 8.9 (8.4–10.4) hours, respectively.

For emtricitabine, *T*_max⁡_ was 1.0 (1.0-2.0) hours in the fasting state and 3.0 (2.0–3.5) hours with food. Emtricitabine *C*_max⁡_ and AUC_0−24_ were significantly lower with food (17% and 13%, resp.) while *C*_24_ was unchanged. In the fasting and fed states, CVs for AUC_0−24_ were 48% and 43%; *C*_max⁡_ was 65% and 74%; *C*_24_ was 57% and 41%, respectively. On Day 1 and Day 8, *t*_1/2_ was 6.1 (5.8–6.8) and 4.8 (4.5–5.7) hours, respectively.

For efavirenz, *T*_max⁡_ was 3.0 (2.0–4.0) hours in the fasting state and 3.0 (3.0–6.0) hours with food. Efavirenz AUC_0−24_ and *C*_max⁡_ were significantly increased in the fed state by 13% and 47%, respectively, while efavirenz *C*_24_ was identical under both meal conditions. High inter-individual variability was observed for *C*_24_ (80% and 85%) in the fasting and fed states, respectively. For these two meal conditions, CV for AUC_0−24_ was 66% and 64% and *C*_max⁡_ was 48% and 45%, respectively. Two individual patients (number 05 and number 10) had unusually high efavirenz concentrations. For these two patients, efavirenz AUC_0-24_ ranged from 119,178 to155,895 ng·h/mL on both sampling visits (Figure 2(c)). These patients were the only two patients with *C*_12_ values above 4000 ng/mL. Their respective *C*_12_ were 4,968 ng/mL and 6067 ng/mL without food and 6,300 and 5936 ng/mL with food. The *C*_24_ measured below 1,000 ng/mL in five patients in the fasting state and four patients in the fed state.

### 3.3. Safety

No study-related adverse events were reported.

## 4. Discussion

Efavirenz trough concentrations were similar under both meal conditions; however, peak concentrations were increased by 47% during administration with a moderate-fat Ugandan meal. However, food did not alter the proportion of patients with efavirenz concentrations above the threshold of 4,000 ng/mL 12 hours after dosing. In 2001, Marzolini et al. reported an approximately 2.5-fold increase in the frequency of sustained central nervous system (CNS) toxicity among patients with efavirenz concentrations above 4,000 ng/mL versus patients with concentrations between 1,000 and 4,000 ng/mL [11]. However, this threshold was not confirmed in a larger analysis which found a correlation between symptoms and plasma levels only in the first week of treatment [12], instead single-nucleotide polymorphisms of drug-metabolizing enzymes were shown to be more predictive of efavirenz pharmacokinetics and clinical outcomes.

Efavirenz is primarily metabolised by hepatic CYP2B6 [7]. In the ACTG A5097s study, a gene-dose effect for efavirenz pharmacokinetics was observed among patients with CYP2B6 polymorphisms with three-fold-higher efavirenz exposure among *CYP2B6 *516T/T homozygotes as compared to G/G homozygotes, and with intermediate values for G/T heterozygotes [13]. A subsequent analysis revealed that the composite *CYP2B6 *516 G→T and 983 T→C genotype best predicted efavirenz pharmacokinetics, suggesting a slow-metaboliser genotype for efavirenz. Among Caucasians, this genotype was associated with a first CNS adverse event (*P* = 0.04). Surprisingly, among Black patients (in whom slow-metaboliser genotypes are more frequent), no association was found [14]. Instead, Black patients with the slow-metaboliser genotype had a lower incidence of virologic failure than other races (*P* = 0.02) [14]. The authors of that study postulated that higher efavirenz concentrations among patients with the slow-metaboliser genotype could permit continued suppression of HIV-1 during episodes of treatment interruption [14].

Therefore, the clinical relevance of the moderately increased efavirenz concentrations observed with food in the current study is a balance between the risk of increased toxicity (at the onset of therapy) and the potential benefit of higher efavirenz exposures leading to a lower incidence of virologic failure, based on the assumption that dosing efavirenz with food would have analogous effects to those seen among Blacks with the slow-metaboliser genotype in the ACTG study.

In the present study, only two patients had efavirenz concentrations in the expected range for patients with the *CYP2B6 *516TT genotype. For other patients, food modestly increased exposure, but absolute concentrations did not attain values of the two patients under either meal condition. Although the genotype of those two patients is not known, one can postulate that genetic influence on efavirenz metabolism plays a more significant role than food on efavirenz pharmacokinetics and efavirenz-related toxicity.

Although the patients in the current study reported no adverse events, formal psychometric testing was not conducted and mild changes in CNS function cannot be completely ruled out. Importantly, these patients had received efavirenz-based therapy for a minimum of seven months prior to enrolment. Since efavirenz-related CNS toxicity tends to resolve within the first few weeks of treatment [7], the safety findings of the current study may not be representative of safety outcomes among patients initiating efavirenz-based regimens. Nevertheless, the findings from the current study suggest that stable patients may administer efavirenz-containing regimens without meal restrictions.

For tenofovir, peak concentrations were unaffected by food intake while tenofovir exposure was marginally increased by food. Emtricitabine exposure and peak concentrations were only mildly reduced (13% and 17%, resp.) by a meal. Tenofovir is a nucleotide reverse transcriptase inhibitor which undergoes intracellular phosphorylation in two steps to its active diphosphate anabolite. Like tenofovir, emtricitabine undergoes intracellular phosphorylation. However, emtricitabine undergoes phosphorylation in three steps to its active triphosphate. For these two drugs, mild and transient changes in plasma concentrations are unlikely to be of clinical relevance as drug effect is not only dependent on absorption and elimination but also on the rate and extent of intracellular phosphorylation [15]. Consequently, TDF and emtricitabine may be taken with or without food.

In general, for lipophilic drugs, absorption is improved by food, particularly food containing fat [16]. Efavirenz is lipophilic, and enhanced absorption with fat is expected. The diester derivative of tenofovir (TDF) was specifically developed to improve the lipophilicity of tenofovir and enhance oral bioavailability [17, 18]; thus, enhanced absorption with fat would also be expected. The increases in tenofovir exposure seen in the current study were of lesser magnitude than those reported with single-dose TDF which may relate to less fat being used in the present study than the single-dose study which had 50% of the calories of a 700–1,000 Kcal meal derived from fat [6]. In contrast to TDF and efavirenz, emtricitabine is an acidic hydrophilic molecule [19]. Fat may interfere with emtricitabine dissolution, and food may delay dissolution of the tablet by reducing gastric pH, resulting in lower emtricitabine exposures with food.

In conclusion, a fat-containing meal moderately increased efavirenz steady-state peak concentrations. In contrast, pharmacokinetic parameters of tenofovir and emtricitabine were mildly affected by food, and those changes do not appear clinically significant. Since efavirenz-related central nervous system toxicity may be concentration dependent, patients experiencing these toxicities should take the FDC tablet without food. However, for patients without toxicities, the FDC can be taken without regard to meals.

## Figures and Tables

**Table 1:** Patient characteristics at screening.

Parameter	Median (interquartile range)
Age (years)	43 (40–50)
Weight (kg)	74 (69–80)
Height (m)	1.70 (1.68–1.75)
CD4 (cells/*μ*L)	355 (312–419)
Hb (g/dL)	14.9 (13.1–15.3)
Alanine transaminase (IU/L)	25 (17–32)
Aspartate transaminase (IU/L)	23 (20–27)
Blood urea nitrogen (mg/dL)	10 (8–13)
Creatinine (mg/dL)	0.87 (0.78–0.94)

**Table 2:** Pharmacokinetic parameters and comparisons during tenofovir and emtricitabine plus efavirenz administration in the fasting (Day 1) and fed (Day 8) states.

	Parameter	GM (95% CI)		GMR (90% CI)
		Fasting*	Fed	Fed/Fasting
	*C*_max⁡_ (ng/mL)	169 (137–255)	175 (136–275)	*1.04 *(0.84–1.27)
Tenofovir	AUC_0−24_ (ng·h/mL)	1316 (1117–1748)	1568 (1369–2032)	**1.19 (1.10–1.29)**
	*C*_24_ (ng/mL)	27 (23–39)	27 (23–34)	*0.99 *(0.82–1.19)
	CL/F_(0−24)_ (L/h)	186 (168–231)	156 (139–198)	*0.84 *(0.78–0.91)

	*C*_max⁡_ (ng/mL)	1043 (935–1233)	870 (789–1016)	**0.83 (0.76–0.92)**
Emtricitabine	AUC_0−24_ (ng·h/mL)	7029 (6300–8313)	6115 (5642–6846)	**0.87 (0.78–0.97)**
	*C*_24_ (ng/mL)	67 (57–92)	61 (53–81)	*0.91 *(0.73–1.14)
	CL/F_(0−24)_ (L/h)	28 (26–33)	33 (30–37)	*0.89 *(0.80–0.99)

	*C*_max⁡_ (ng/mL)	3128 (2678–4203)	4611 (3961–6037)	**1.47 (1.24–1.75)**
Efavirenz	AUC_0−24_ (ng·h/mL)	46299 (37411–69475)	52194 (41827–76643)	*1.13 (1.03–1.23)*
	*C*_24_ (ng/mL)	1395 (1082–2335)	1408 (1073–2448)	*1.01 (0.91–1.11)*
	CL/F_(0−24)_ (L/h)	13 (11.6–17.3)	12 (10.5–14.4)	**0.89 (0.81–0.97)**

GM: geometric means; GMR: geometric mean ratio; CI: confidence intervals, *C*_max⁡_: maximum concentration; *C*_24_: concentration 24 hours after dosing; AUC_0−24_: area under the concentration-time curve; CL/F: clearance.

*reference.
